# Revealing the stimulus-driven component of attention through modulations of auditory salience by timbre attributes

**DOI:** 10.1038/s41598-023-33496-2

**Published:** 2023-04-26

**Authors:** Baptiste Bouvier, Patrick Susini, Catherine Marquis-Favre, Nicolas Misdariis

**Affiliations:** 1 STMS IRCAM, Sorbonne Université, CNRS, Ministère de La Culture, 75004 Paris, France; 2 Univ Lyon, ENTPE, École Centrale de Lyon, CNRS, LTDS, UMR5513, 69518 Vaulx-en-Velin, France

**Keywords:** Human behaviour, Acoustics

## Abstract

Attention allows the listener to select relevant information from their environment, and disregard what is irrelevant. However, irrelevant stimuli sometimes manage to capture it and stand out from a scene because of bottom-up processes driven by salient stimuli. This attentional capture effect was observed using an implicit approach based on the additional singleton paradigm. In the auditory domain, it was shown that sound attributes such as intensity and frequency tend to capture attention during auditory search (cost to performance) for targets defined on a different dimension such as duration. In the present study, the authors examined whether a similar phenomenon occurs for attributes of timbre such as brightness (related to the spectral centroid) and roughness (related the amplitude modulation depth). More specifically, we revealed the relationship between the variations of these attributes and the magnitude of the attentional capture effect. In experiment 1, the occurrence of a brighter sound (higher spectral centroid) embedded in sequences of successive tones produced significant search costs. In experiments 2 and 3, different values of brightness and roughness confirmed that attention capture is monotonically driven by the sound features. In experiment 4, the effect was found to be symmetrical: positive or negative, the same difference in brightness had the same negative effect on performance. Experiment 5 suggested that the effect produced by the variations of the two attributes is additive. This work provides a methodology for quantifying the bottom-up component of attention and brings new insights on attention capture and auditory salience.

## Introduction

The acoustic environment is so rich in information that our brain cannot process in detail all of the sounds it is constantly receiving. Instead, the individual selects stimuli that they deem to be relevant for a particular task, and ignores others^1^. The most famous example of selective attention is the cocktail party problem^2^. This ability is made possible by an attentional process that filters the flow of stimulus information through certain irrelevant channels^3,4^. The precise mechanisms involved in this filtering are still being investigated^5^. However, the brain should not be completely blind to task-irrelevant stimuli since they could provide important information about the environment. For example, if we are chatting to someone on the street, we can pick up what they are saying and ignore the surrounding traffic noise. However, the squeal of tires associated with a car's sudden braking may still attract our attention. So, if the stimulus is sufficiently salient, the brain may have to process the information it contains involuntarily. This phenomenon is known as involuntary attentional capture. Salience is the property of a stimulus that makes it likely to capture attention, i.e., the bottom-up component of attention^6^.

Attention capture has been extensively studied in the visual modality (see 30 for a review). Implicit approaches measure the behavioral costs (increased reaction times and error rates) of the presence of an irrelevant distractor in focal tasks. Among other things, irrelevant stimuli defined by their color, shape or onset time are known to attract the attention of participants performing a visual search task^7–9^.

However, there has been some debate about how salient objects can automatically capture attention. Some have argued that salient objects have an automatic power to attract attention, regardless of the subject's goals. They observed that certain features, such as color or shape, make the salient object automatically capable of attracting attention^10^. This led to a stimulus-driven conception of attentional capture^11^: visual selection is determined by the physical properties of the stimuli, and attention is drawn to the location where one object differs from the others along a particular dimension. However, others have argued that only items that match the target's features can capture attention. For them, capture depends on the attentional set that is encouraged by the task^12^. For example, it has recently been found that salience does not influence the capture of visual stimuli. Instead, participants can often learn to suppress salient objects^13,14^. Authors from different parties eventually came together to review and compare their theories^15^. They agreed that "physically salient stimuli automatically generate a priority signal that, in the absence of specific attentional control settings, will automatically capture attention, but there are circumstances under which the actual capture of attention can be prevented", reconciling the stimulus-driven and contingent capture approaches.

In the auditory modality, few studies have addressed this issue. Huang and Elhilali^16^ used an explicit approach to measure auditory salience in complex sound scenes. Participants listened to the scenes dichotically (a different scene in each ear), and continuously indicated which side their attention was focused on. Averaged across scenes and participants, this allows the identification of salient events in a scene where their responses, on average, indicate how they orient their attention. This protocol involves top-down processes, as participants actively listen to the sounds and report the orientation of their attention. We therefore cannot infer any measurement of the purely bottom-up component of attention. In Kaya et al.^17^ the authors asked their participants to focus on a visual task and to ignore background acoustic melodies. Brain responses were recorded, showing that variations in acoustic attributes could make notes in these melodies more salient, and how these different attributes interacted to modulate brain responses.

Dalton and Lavie^18^ used an implicit approach based on the additional singleton paradigm to reveal an auditory attentional capture effect by sound features such as frequency or intensity. This paradigm was first developed in the visual modality to show that irrelevant stimuli can capture participants' attention during a visual search task, resulting in increased error rates and response times^7,19^.

Results from Dalton and Lavie^18^ showed a significant cost (increased response times and error rates) in an auditory search task caused by irrelevant sounds. In their experiment, participants had to listen to sequences of five sounds. Among these, they had to detect a target defined by a dimension (e.g., a change in frequency compared to non-targets). In half of the trials, one of the non-targets was made different from the others on a dimension other than that which defined the target, such as intensity. This sound is called a singleton and is irrelevant to the task. In fact, paying attention to the dimension that defines the singleton is not an advantageous strategy for detecting the target. The results showed that the singleton features could cause interference: participants made more errors and took more time to detect the target when the singleton was present. The effect was not due to low-level interactions between the singleton and the target, which would have caused it to be more difficult to compare the target with the singleton than with a non-target. The effect was shown when the singleton was separated from the target by another sound. Garrido et al.^20^ discussed the similarity to mismatch negativity studies, which focus on the elicitation of an event-related potential by deviant tones that differ in frequency or duration. The much shorter inter-stimulus interval, the frequency of occurrence of the deviant tones, and the explicit instruction to ignore these irrelevant singletons limit the parallels that can be drawn in this area of research. Dalton and Lavie^18^ focused on the attentional capture produced by singletons of different frequency or intensity, but did not investigate the effects of sounds whose features are gradually modified.

In addition, the study of variations in intensity, and therefore loudness, of sounds may be compromised in this paradigm. Masking effects are likely to occur for louder sounds and interfere with the attentional processes we wish to study^21^. However, the paradigm is compatible with the study of variations in timbre. One precaution would be to equalize all sounds in loudness to remove potential masking effects and the influence of loudness, which can be affected by pitch or timbre variations^22^.

None of the approaches mentioned here focused on the relationship that may exist between variations in the acoustic attributes and the attentional capture effect.

The first acoustic feature one might think of when studying salience is loudness. Sounds that are perceived as louder are more likely to attract the listener’s attention. Loudness has been shown to be an important feature of salience^16,23,24^. In addition to this feature, several studies have shown that some dimensions of timbre can be sound markers for conveying relevant information. Lemaitre et al.^25^ found that listeners used common perceptual dimensions to categorize car horns. Two of the three dimensions identified were roughness and brightness. Arnal et al.^26^ noted that amplitude modulated sounds in the roughness range are found in both natural and artificial alarm signals, and are better detected due to the privileged space they occupy in the communication landscape. Rough sounds are also said to enhance aversiveness through specific neural processing^27^. Brightness has long been known to be a major dimension of musical timbre^28^ and has therefore been included in most salience models^16,29^. More recently, roughness has also been included^30^.

Thus, the existence of the stimulus-driven component of attention capture has been theoretically established. Moreover, the additional singleton paradigm allows the measurement of the attentional capture effect due to sound features. Finally, the literature findings suggest that certain attributes of the sound timbre are potential candidates that could be responsible for the salience of a sound, and thus its ability to capture attention. However, to the authors’ knowledge, no study has ever established the relationship that might exist between variations in these features and the magnitude of the attentional capture effect. In other words, the driving properties of attentional capture by the stimulus features have not yet been revealed.

In the present work, we adopted the additional singleton paradigm to provide evidence for the effect of timbre features on attentional capture. We then used this paradigm to quantify the relationship that may exist between a sound feature and the associated capture effect. Thus, in the current study, we focused on the properties of the *stimulus-driving* of the attentional capture effect.

To summarize, we wanted to answer the two following questions:

- Do timbre attributes such as brightness or roughness trigger attention capture?
- How do their variations drive attention capture?

First, the possibility of an attentional capture by a timbre variation was investigated. Therefore, the spectral centroid (SC) of the singleton, which correlates with its perceived brightness, was investigated in experiment 1. Then, the same experimental procedure was used to evaluate how the effect size was modulated by feature variations. In experiments 2 and 3, the SC and the depth of amplitude modulation (correlated with roughness) could take several different values. Finally, experiment 4 examined the effect of symmetric variations in brightness and experiment 5 focused on combined variations in brightness and roughness to investigate the directionality and additivity of attentional modulation.

## Experiment 1: attentional capture by a bright singleton

### Method

#### Transparency and openness

We report how we determined our sample size, all data exclusions, all manipulations and all measures in the study. Data were collected in 2021 and 2022 and analyzed using python 3.7. All statistical analyses were performed using python 3.7 and the open-source pingouin package.

#### Participants

A previous pilot experiment involving 11 participants was conducted to calculate the power of the effect of the singleton presence on response time. The calculus was made for a one-tailed t-test, with an effect size of d = 0.8, α = 0.05 and aiming for a power of 0.8, and determined a minimum sample size N = 12.

Thus, 15 participants (8 females, 7 males) took part in this experiment. They ranged in age from 20 to 45 years (mean age: 31 ± 8 years). They were all consenting and reported normal hearing. An audiometry in the frequency range between 0.125 and 8 kHz was performed for each participant and revealed no hearing impairment. The protocol was approved according to Helsinki Declaration by the Ethics Committee of *Institut Européen d’Administration des Affaires* (INSEAD). All methods were carried out in accordance with their guidelines and regulations. Participants gave written informed consent and received financial compensation for their participation.

#### Apparatus

The experiment was designed and run on Max software (version 7, https://cycling74.com), on a Mac mini 2014 (OS Big Sur 11.2.3). The stimuli were designed with python 3.7, and presented during the experiment through headphones (Beyerdynamic 770 pro, 250 Ohm). The experiment took place in the STMS laboratory of IRCAM in a soundproofed double-walled IAC booth.

#### Stimuli

**Figure 1:**
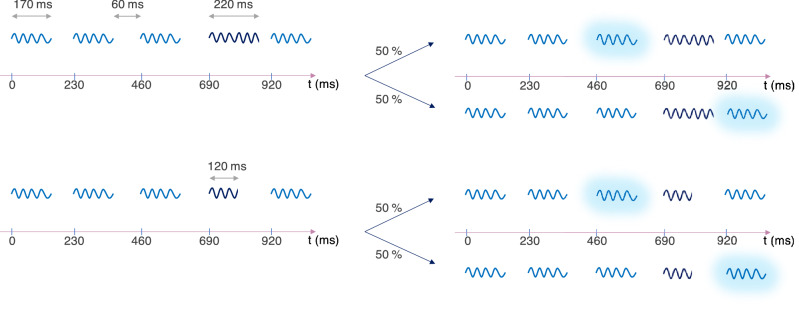
Stimuli without (left) and with (right) a singleton (surrounded with a glow), with 50% chances being before or after the target (dark blue). Only sequences with target in position 4 are shown here.

The stimuli were made of sequences of 5 sounds (see Fig. 1). All notes follow the harmonic structure of Bouvier et al.^31^, with 20 harmonics, the n^th^ harmonic f_n_ having a frequency n*f_0_ and a weight $\frac{1}{{n}^{\mathrm{\alpha }}}$. Thus, decreasing α increased the sound spectral centroid (SC), and therefore its perceived brightness: $SC= \frac{\sum_{i=1}^{20}\frac{fi}{{i}^{\mathrm{\alpha }}}}{\sum_{i=1}^{20}\frac{1}{{i}^{\mathrm{\alpha }}}}$ .

##### Distractor

For the reference distractor, α = 3. It lasted 170 ms, with a ramp at the beginning and end of 5 ms, and had a SC equal to 512 Hz.

##### Targets

The targets were 50 ms shorter or longer than the distractor. This value is higher than what Abel^32^ found as a just-noticeable difference (jnd) for duration discrimination of sinusoidal sounds. Based on previous tests done in the lab, the experimenters still ensured beforehand that the targets were clearly heard as distinct from the distractors. The targets had the same fundamental frequency and spectrum distribution (α = 3) as the reference distractor, but a duration of 220 ms for the long one and 120 ms for the short one.

##### Singleton

The singleton had the same fundamental frequency and envelope as the reference distractor, but a different spectrum distribution with α = 2. It resulted in a higher SC, equal to 822 Hz. Allen and Oxenham^33^ found a jnd of 5.0% for the SC, which ensures the singleton was indeed perceived brighter. The experimenters still ensured beforehand that the singleton was clearly heard as distinct from the distractors.

In the reference condition, the target was embedded in sequences of distractors only such that a sequence was composed of four distractors and a target stimulus. In the test condition, one of the distractors was the singleton such that a sequence was composed of three distractors, one target and one singleton. The IOI ("Inter-Onset Interval") was kept constant at 230 ms. The first sound of each sequence was always a distractor. The target was in 3rd or 4th position (50% of the trials each). In the trials containing a singleton, its position was either just before or just after the target (50% of the trials each). All the conditions are presented in Fig. 1.

#### Loudness equalization

All the sounds were equalized in an adjustment experiment with 12 participants from the lab, using same setup as the main experiment. Loudness adjustments were performed by comparing all the sounds (short target, long target or singleton) to a reference (the distractor presented at 80 dB SPL). The sounds were randomly distributed and presented 8 times each. The levels were measured at the headphones output with a Brüel and Kjaer 2238 mediator sound level meter. The obtained levels were 81 dB SPL for the short target, 79 dB SPL for the long target and 74 dB SPL for the singleton. All inter-participants standard deviations of these obtained levels were less than 1 dB SPL, i.e., less than a just-noticeable difference in sound level^34^.

#### Procedure

Six blocks of 60 randomly distributed trials were run for each participant. For every trial, the word *Ready* was displayed on the screen for 1500 ms, then a sequence of 5 sounds was presented.

At the end of the sequence, the participant could respond by pressing a keyboard: "1" for "short" and "2" for long (2 alternative forced choice protocol). Feedback regarding the participant’s response (*Correct* or *Incorrect*) was displayed after each trial and remained for 1500 ms. If after 3000 ms no answer was given by the participant, the message *Too late. Answer faster!* was displayed. The response time was measured from the moment the target was played in the sequence. Then, a 1500 ms pause occurred and the next trial began.

The participants were asked, at the beginning of the experiment, to focus on the duration of the sounds and their duration only in order to discriminate the target. Each participant had a training block before taking the test. We kept only the results of participants with an error rate below 40% on the sequences containing the target. Due to this criterion, one participant had to be replaced at this step. The experiment lasted 90 min on average.

### Results

For each participant, and for each singleton condition (absent or present), we calculated the mean and the standard deviation of the response times. We then removed the data whose response time was more than two standard deviations from the mean^35^. We also removed the data for which the response time was less than 100 ms, and those for which the participant did not answer. 94.9% of the data were kept at this stage. For the response time analysis, only the data where the participant's response was correct were kept, i.e., 75.6% of the data. The results of mean error rates and response times are presented in Table 1. For all the following experiments, error rates follow the same trends as response time increases. The LISAS (Linear Integrated Speed Accuracy Score—^36^) were also computed and followed the same trends. For the sake of clarity, we therefore show only the increases in response time.

**Table 1:** Mean and standard deviation of response times and error rates (across the 15 participants) depending on the presence of the bright singleton.

Singleton	Absent	Present
Response time (Standard deviation)	985 ms (142)	1121 ms (185)
Error rate (Standard deviation)	16.2% (13.5)	24.2% (15.1)

The error rates (16.2% and 24.2% in the conditions without and with a singleton, respectively) confirm that participants were able to complete the task correctly in both conditions. The mean response time increase, when the singleton was present, was 137 ms. A t test revealed that the singleton presence had a significant effect on response time increase (t test: t(14) = 8.33, *p* < 0.001). The effect of the singleton presence was very large (cohen-d = 2.1). A very large effect of the singleton presence was found for error rates as well (t(14) = 3.85, *p* < 0.001, cohen-d = 1.0).

The effect of the singleton position on error rates was not significant (t(14) = 0.72, *p* = 0.48), suggesting that attentional capture occurs as much whether the singleton appears before or after the target. However, there was an effect of the singleton position on response times (t(14) = 4.38, p < 0.001): when the singleton appeared after the target, the response times were greater. This absence of effect of the singleton position on error rates and the increased reaction times when the singleton occurs after compared to before the target confirm that this effect is not due to auditory masking. This is consistent with the loudness equalization that had been carried out beforehand and the IOI which prevented auditory masking^21^. The observed effect is due to an attentional capture caused by the bright singleton. Finally, one could claim that the effect is due to the surprise caused by the occurrence of the singleton. However, this singleton is present in 50% of the trials, and the participants identified and accustomed themselves to it during the training session. Moreover, no significant difference was found for response times between trials where a singleton appears after one or more trials without any singleton (the "surprising" condition), and trials where the singleton is present after one or more trials with a singleton (the "non-surprising" condition): t(14) = 0.31, *p* = 0.76.

This first experiment thus allowed us to validate the framework in which we can test modulations of timbre features and observe how they drive the attentional capture effect. It was therefore decided to reproduce the experiment, modifying it so that the singleton could take different values of brightness in a second experiment, and different values of roughness in a third one.

## Experiments 2 and 3: variations of brightness and roughness

Experiments 2 and 3 were conducted to study how the effect magnitude is modulated by the singleton feature variations. In experiment 2, we replicated experiment 1 with four different values of the spectral centroid (SC) for the singleton. In experiment 3, four values of the amplitude modulation depth for the singleton were used. This latter sound feature is associated to an auditory attribute usually described by the semantic attribute “roughness”^37^.

### Method

#### Participants

Twenty participants (10 females, 10 males) took part in experiment 2, and 20 others (10 females, 10 males) in experiment 3. The sample size was increased to ensure that the power of the effect produced by the second-brightest singleton was greater than 0.8. This was done in order to have at least two different brightness conditions with sufficient power. The participants ranged in age from 19 to 34 years (mean age: 27 ± 4 years) for experiment 2, and from 22 to 50 years (mean age: 28 ± 8 years) for experiment 3. They were all consenting and reported normal hearing. An audiometry in the frequency range between 0.125 and 8 kHz was performed for each participant and revealed no hearing impairment. Participants gave written informed consent and received financial compensation for their participation.

#### Apparatus

The apparatus was the same as in the first experiment, except that it took place in the INSEAD-Sorbonne Université Behavioural Lab, in soundproofed rooms.

#### Stimuli

The distractors and targets were the same as in experiment 1. For experiment 2, the singleton SC could take 4 values: 538, 563, 640 or 768 Hz. Each one was presented in 20% of the trials. To establish these values, an increment of SC was calculated (using the estimation of 5% for SC jnd found by Allen and Oxenham^33^, and then multiplied by 1, 2, 5 and 10. For experiment 3, the singleton signal s_sing_(t) was the distractor signal s_dis_(t) modulated at a modulation frequency f_mod_ = 50 Hz: ${s}_{sing}\left(t\right)=\left(1+m*\mathrm{cos}\left(2\pi {f}_{mod}t\right)\right)*{s}_{dis}\left(t\right).$ The modulation depth m could take 4 values: 0.1, 0.2, 0.5 or 1.0. Each one was presented in 20% of the trials. To establish these values, the increment of modulation depth estimation proposed by Zwicker and Fastl^37^ (10%) was multiplied by 1, 2, 5 and 10 as well.

#### Loudness equalization

The loudness of the singletons was equalized as in experiment 1. The levels obtained for each singleton after equalization were 79.5, 79.0, 77.5 and 75.0 dB SPL for the bright singletons with SC of 538, 563, 640 and 768 Hz, respectively, and 80 dB SPL for all the rough singletons. All inter-participants standard deviations of the obtained levels were less than 1 dB SPL.

#### Procedure

The procedure was the same as in experiment 1, except that the number of trials had to be increased because of the increased number of singletons. Eight blocks of 80 randomly distributed trials each were run for each participant.

### Results

**Figure 2:**
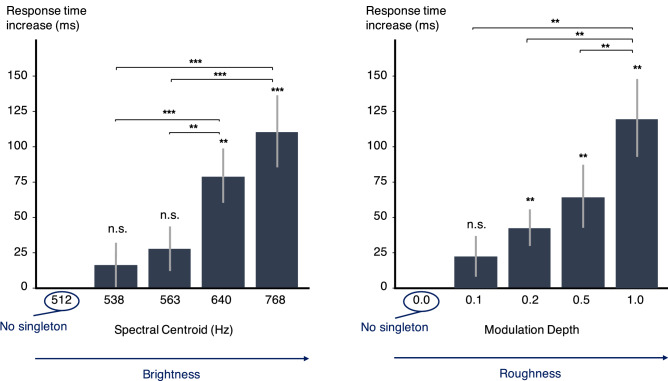
Increase in response time (ms) with singleton SC (left, experiment 2) and modulation depth (right, experiment 3). Error bars represent the standard errors across participants in each condition compared to the no-singleton condition. Significances between conditions are displayed on the horizontal braces. *: *p* < .05, **: *p* < .01, ***: *p* < .001.

The data processing was the same as for experiment 1. For the error rate analysis, 95.0% and 94.6% of the data were kept for experiments 2 and 3, respectively. For the response time analysis, only the data where the participant's response was correct were kept, i.e.*,* 78.6% and 76.4% of the data. The mean response time and error rate across the 20 participants for sequences without singleton were 867 ms (std = 246 ms) and 12.6% (std = 13.1%) for experiment 2, 1058 ms (std = 294 ms) and 15.2% (std = 12%) for experiment 3. The increase in response time for each singleton, i.e.*,* the difference between the condition with the considered singleton and the reference condition without any singleton, is presented in Fig. 2 for each value of modulation depth and spectral centroid.

For both experiments 2 and 3, t-tests were conducted with Holm corrections for repeating comparisons. Complete statistics can be found in the Supplementary information (S1 and S2).

Data from experiment 2 confirmed and extended the result of experiment 1 as various bright singletons produced an attentional capture effect. Moreover, the effect increased with SC values: the brighter the singleton, the greater the effect. Experiment 3 showed that roughness is also a feature that triggers an attentional capture effect: the presence of various rough singletons caused significant behavioral costs. The results confirmed that there is a dependency of salience with the variations of the feature which define the singleton.

Interestingly, the manipulations of the two timbre attributes resulted in comparable effect magnitudes. An increase of a few increments on brightness gives an effect similar to that obtained with an increase of the same number of increments on roughness. This is discussed in the general discussion.

## Experiment 4: symmetrical variations of brightness

Experiment 4 was conducted to study the symmetry or the directionality of the effect. We replicated experiment 2 with SC values for the singleton being either higher or lower than the distractors SC.

### Method

#### Participants

19 participants (8 females, 11 males) took part in the experiment 4. They ranged in age from 18 to 32 years (mean age: 25 ± 4 years). They were all consenting and reported normal hearing. An audiometry in the frequency range between 0.125 and 8 kHz was performed for each participant and revealed no hearing impairment. Participants gave written informed consent and received financial compensation for their participation.

#### Apparatus

The apparatus was the same as in the first experiment, except that it took place in the INSEAD-Sorbonne Université Behavioural Lab, in soundproofed rooms.

#### Stimuli

The distractor and target SC was equal to 631 Hz. The singleton SC was 2 and 4 jnd higher or lower than the distractor one, i.e.*,* 512, 569, 696 or 768 Hz. Each one was presented in 20% of the trials. All the sounds were equalized in loudness (12 participants with the same procedure as in experiment 1): the obtained levels were 80, 79, 77 and 75 dB SPL for the singletons with SC at 512, 569, 696 and 768 Hz respectively, and 78 dB SPL for the distractor. All inter-participants standard deviations were less than 1 dB SPL.

### Results

**Figure 3:**
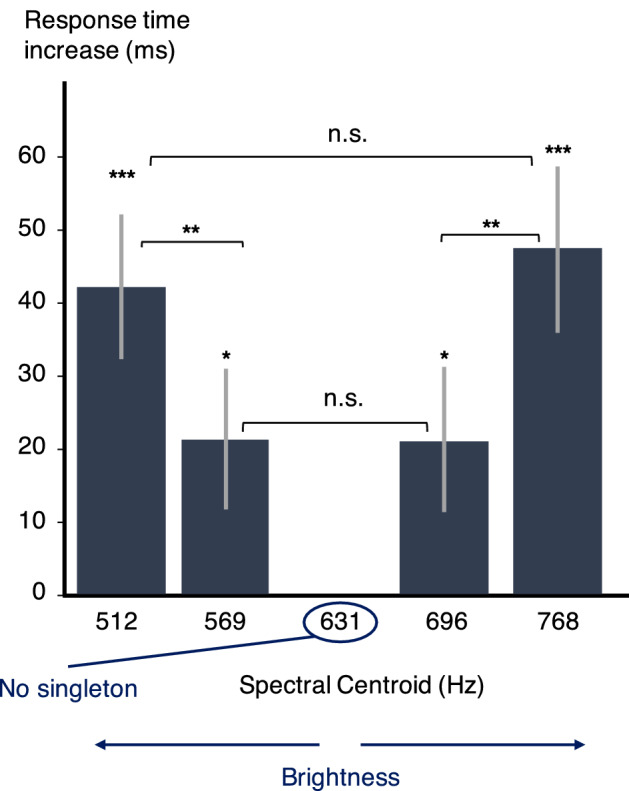
Increase in response time (ms) with singleton SC (experiment 4). Error bars represent the standard errors across participants in each condition compared to the no-singleton condition. Significances between conditions are displayed on the horizontal braces. *: *p* < .05, **: *p* < .01, ***: *p* < .001.

The data processing was the same as for experiment 1. For the error rate analysis, 94.7% of the data were kept. For the response time analysis, only the data where the participant's response was correct were kept, i.e.*,* 87.1% of the data. The mean response time and error rate across the 19 participants for sequences without singleton were 940 ms (std = 195 ms) and 5.1% (std =  ± 8.4%). The increase in response time for each singleton, i.e.*,* the difference between the condition with the considered singleton and the reference condition without any singleton, is presented in Fig. 3. Complete statistics can be found in the Supplementary information (S3).

**Figure 4:**
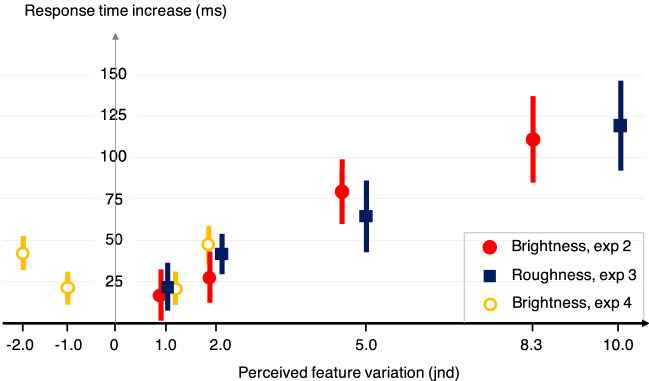
Increase in response time (ms) depending on the singleton perceived feature variations (jnd) in experiments 2, 3, and 4. Error bars represent the standard errors across participants in each condition compared to the no-singleton condition.

The effect magnitudes are comparable to those obtained in experiment 2. A clear symmetry is observed in experiment 4: the effect of a brighter singleton is the same as the one of a less bright singleton, if both vary absolutely by the same amount of perceived brightness. This result tells us that it is the absolute variation of the singleton feature that modulates the attention capture. The results of experiments 1, 2, 3 and 4 can be summarized in Fig. 4, which shows the driving of response time increases by the perceived variations in the singleton feature. These perceived variations are shown in terms of jnd values.

Interestingly, a linear relationship seems to emerge between increases of perceived brightness (combined across experiment 1, 2 and the positive variations in experiment 4) and response time increase (r_Pearson_(3) = 0.99, *p* < 0.001, slope = 14.0 ms—std error = 0.9 ms), and for perceived roughness as well (r_Pearson_(3) = 0.99, *p* < 0.01, slope = 12.4 ms—std error = 0.9 ms). This relationship is only valid for this range of feature variations and is discussed in the general discussion.

## Experiment 5: combination of roughness and brightness

Experiment 5 was conducted to study the additivity of the effects of different features variations. We replicated experiment 2 with four different singletons, having different combinations of roughness and brightness. The singleton could have two different SC combined with two different amplitude modulation depths.

### Method

#### Participants

Nineteen participants (9 females, 10 males) took part in the experiment 4, whose ages ranged from 21 to 36 years (mean age: 26 ± 5 years). They were all consenting and reported normal hearing. An audiometry in the frequency range between 0.125 and 8 kHz was performed for each participant and revealed no hearing impairment. Participants gave written informed consent and received financial compensation for their participation.

#### Apparatus

The apparatus was the same as in the first experiment, except that it took place in the INSEAD-Sorbonne Université Behavioural Lab, in soundproofed rooms.

#### Stimuli

The distractor and target SC was equal to 512 Hz, and they were not modulated, i.e., null roughness. The singleton SC was 2 or 5 jnd higher than the distractor one, i.e.*,* 564 and 653 Hz. The singleton modulation depth was 2 or 5 jnd higher as well, i.e.*,* 0.2 and 0.5. The four singletons were thus obtained with the four combinations of these SC and modulation depths. Each one was presented in 20% of the trials. All the sounds were equalized in loudness (12 participants with the same procedure as the one used in experiment 1): the obtained levels were 79 dB SPL for the singletons with 2 jnds of brightness, 77.5 dB SPL for the singletons with 5 jnds of brightness. All inter-participants standard deviations were less than 1 dB SPL.

### Results

**Figure 5:**
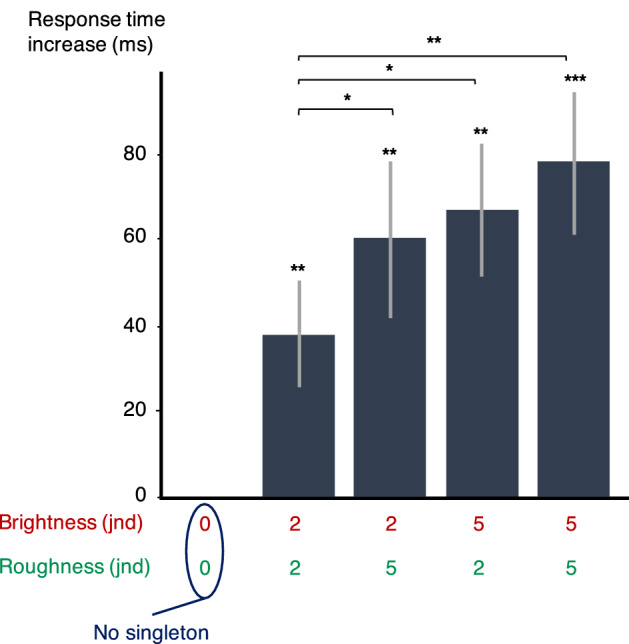
Increase in response time (ms) with singleton SC and modulation depth (experiment 5). Error bars represent the standard errors across participants in each condition compared to the no-singleton condition. Significances between conditions are displayed on the horizontal braces. *: *p* < .05, **: *p* < .01, ***: *p* < .001.

The data processing was the same as for experiment 1. For the error rate analysis, 94.9% of the data were kept. For the response time analysis, only the data where the participant's response was correct were kept, i.e.*,* 74.9% of the data. The mean response time and error rate across the 19 participants for sequences without singleton were 994 ms (std = 158 ms) and 17.4% (std = 12.7%). The increase in response time for each singleton, i.e.*,* the difference between the condition with the considered singleton and the reference condition without any singleton, is presented in Fig. 5. Complete statistics can be found in the Supplementary information (S4).

The effect produced by a 2 + 2-jnds variation here is comparable to that produced by a 2-jnds variation in experiments 2 and 3. It is uncertain whether this is due to a non-additivity of the effects of the combined features or whether participants were simply less subject to attentional capture in this experiment. Nevertheless, within their range of magnitudes, the response times in experiment 5 appear to increase linearly with the addition of the perceptual variations on the two dimensions (r_Pearson_(3) = 0.99, p < 0.01, slope = 8.5 ms—std error = 0.4 ms). In other words, the effect seems to be additive across dimensions in this range of values.

### Public significance statement

These findings provide evidence that the perception of certain auditory features drives the ability of sounds to capture our attention, according to laws that are revealed.

## General discussion

Results from experiment 1 showed that a singleton defined by its timbre, specifically its brightness, captured participants’ attention despite being irrelevant to the task they had to perform. Experiment 2 proved that the effect magnitude was driven by the singleton brightness. Experiment 3 showed that a different attribute, roughness, also drives the attentional capture effect. Results from experiment 4 and 5 revealed that this effect is symmetrical, i.e., that only the absolute perceived deviation matters, and additive, i.e., that combining features produces the addition of the effects that each feature variation produces alone.

Thus, in a series of 4 different experiments (2, 3, 4 and 5), a driving of attentional capture by the singleton feature was observed. All else being equal in the experiments, the participants' attentional state remained identical across the different values of the singleton feature. Nevertheless, the magnitude of the effect increased with increasing brightness or roughness variation. The results cannot be explained by increasing singleton-target similarity, because the timbre variations defining the singletons did not make them more similar to the target. Since the increased response times cannot be explained by top-down processes that change with the value of the singleton feature, the observed relationships represent purely feature-driven components of the effect. In other words, the bottom-up component of the attentional capture effect is revealed here, not only confirming its existence^11^, but also revealing its pattern.

Thus, by varying the timbre of the tones while keeping the participants' attentional state fixed, we were able to elicit only the bottom-up component of attentional capture. However, the nature of our protocol itself could raise questions about the participants' attentional state and thus the origin of the capture. The contingency on participants' attentional state^12^ is questionable here. Indeed, according to the contingency hypothesis^15^, the task leads to an attentional state that favors the detection of singletons, and this is why attention is captured by the singleton. However, in the present experiments, there were two single items (out of five) in 80% of the trials, and the singleton was one out of 4 possible singletons. Furthermore, all sounds had a fundamental frequency randomly drawn from a broad uniform distribution of 20 Hz. Thus, the variability of the items was increased in our protocol, and the target was not a single item among all identical items. The single-item detection strategy may therefore no longer be advantageous in this setting, and the adaptation of the singleton to participants' attentional state may be different from that which was traditionally thought to be responsible for detection in this paradigm. Further work is needed to understand the interactions between the bottom-up component revealed here and top-down processes, and to address the issue of the compatibility of these results with the contingent capture approach. For example, it would be important to investigate how the driving by the singleton features evolves as participants change their attentional state.

The feature-driven relationships obtained make it possible to observe and compare how different features modulate attention capture. Indeed, the marginal increase of the effect (the derivative of the curves of response time increases with the perceptual variations of the feature) can be interpreted as the weight of the feature in the sound salience. Interestingly, in experiments 2 and 3, both features drove the effect in a similar way. Either these two features are by chance equally responsible for the salience of a sound, or it is the perceived deviation on each dimension that is important in making a sound salient. This evolution of attentional capture with variations of different features therefore deserves to be confirmed through more experiments involving more features (harmonicity, attack time, spectral flux…). If a similar driving is found for other features, it would show that it is precisely how different the sound is perceived that matters to trigger attentional capture, regardless of the feature used. On the contrary, some features could drive the effect with more or less power. This would lead to a hierarchy of features that influence the salience of a stimulus in terms of its ability to capture attention.

Furthermore, the combined results of experiments 1, 2, 3 and 4 (summarized in Fig. 4) reveal a monotonic relationship between the perceived difference of the singleton feature (quantified in just-noticeable differences) and the increase in reaction time. Thus, the attentional capture effect increases progressively with the perceived difference, according to a law that appears to be linear in the range of deviations tested. This law cannot extend over a very wide range of values, as the capture effect must saturate at some point. In any case, we observe that there is no threshold effect, the function is monotonic and continuous. A more precise and extensive determination of this function could also be further investigated in future studies.

This work also brings new insights into the understanding of auditory salience itself, confirming the importance of timbre in this property. Both brightness and roughness were found to be responsible for an attentional capture by irrelevant sounds. It therefore appears that timbre is also a key dimension in directing auditory attention, in addition to the main dimensions of frequency and intensity highlighted by Dalton and Lavie^18^. The results on brightness confirm the findings that previously led some researchers to consider this feature in their salience model^16,29^. Roughness has only recently been included in some form: Kothinti et al.^30^, for example, added average fast temporal modulations to the latest version of their model. The relationship found between attentional capture and feature variations seems to be supported by both features and deserves further investigation, either in other contexts (other tasks, more complex environments…) or with other features.

Our results show that attention capture is driven by absolute deviations of the sound features. In other words, the features do not have an intrinsic polarity with respect to salience (e.g., the brighter, the more salient). Rather, it is a dissimilarity effect that modulates it. This is consistent with predictive coding and theories of auditory deviance detection^38^. They suggest that the deviations between the prediction and what is subsequently perceived determine auditory salience and trigger notified events^39,40^. Here, we support these theories by showing that absolute deviations of the sound features directly modulate the magnitude of the attentional capture effect, i.e., their salience.

Finally, our findings are interesting from the perspective of auditory salience modelling, which could be improved by knowing the relevant parameters to consider and how salience depends on their variations. The approach taken so far is to consider the absolute and normalized feature variations over time^16,39,41^, without implying a more elaborate modulation of attention with these variations. The additivity of the effect produced by different feature variations provides insights into how to combine them^41^. An interesting avenue might be to consider more complex interactions and to go deeper in the understanding of the mechanisms underlying auditory salience.

## Conclusion

This work provides contributions on a theoretical, methodological and practical level. From a theoretical point of view, a driving of attention capture by a stimulus feature was revealed. This modulation of bottom-up attention was found to be monotonic and similar for the two timbre attributes studied here: brightness and roughness. The experiment with variations in brightness highlighted symmetric properties, and the experiment with combinations of both attributes underlined the non-additive character. Methodologically, a way to measure the feature-driven component of attention was proposed: it implies modulating the singleton features in an additional singleton paradigm while keeping the attentional state constant. From a practical perspective, the results may enrich salience models that can include these features and the way they modulate salience in their implementation.

Finally, this study opens perspectives and calls for further studies. The extendibility of the modulation law to more features and to a wider range of feature variations, its dependence on attentional sets and top-down processes, and a higher resolution of the modulation curves deserve further investigation.

## Supplementary Information

Supplementary Information.

## Data Availability

All data are available at https://github.com/BouvierBaptiste/Revealing-the-stimulus-driven-component-of-attention-through-modulations-of-auditory-salience-by-tim.git.
