# Supplementary material for: Revealing the stimulus-driven component of attention through modulations of auditory salience by timbre attributes
Source: Sci Rep. 2023 Apr 26;13:6842. doi: 10.1038/s41598-023-33496-2 (PMC10133446; doi:10.1038/s41598-023-33496-2)
Supplement: Supplementary file 1 — Supplementary Information. [file 41598_2023_33496_MOESM1_ESM.pdf]

Supplementary information: statistics

Brightness deviation (jnd)	1	1.9	4.6	8.3
p	0.14	0.04	<.001	<.001
p_adjusted	0.14	0.08	.001	<.001
cohen-d	0.24	0.41	0.93	0.97

S1: effect significance (p), adjusted significance (p_adjusted, with Holm corrections for repeating comparisons) and size (cohen-d) of t-tests on the response time increases depending on the different singleton brightness values (quantified in jnd) in experiment 2.

Roughness deviation (jnd)	1	2	5	10
p	0.07	0.001	0.003	<.001
p_adjusted	0.07	0.006	0.008	0.001
cohen-d	0.35	0.76	_	_

S2: effect significance, adjusted significance (Holm corrections for repeating comparisons) and size of t-tests on the response time increases depending on the presence of the different singleton roughness values (quantified in jnd) in experiment 3. The shaded columns correspond to conditions for which the distribution is not normal (revealed through a Shapiro test), and for which Wilcoxon tests were applied.

Brightness deviation (jnd)	-4	-2	2	4
p	<.001	0.02	0.03	<.001
p_adjusted	0.002	0.04	0.04	0.002
cohen-d	0.93	0.49	0.48	0.93

S3: effect significance, adjusted significance (Holm corrections for repeating comparisons) and size of t-tests on the response time increases depending on the different singleton brightness values (quantified in jnd) in experiment 4.

[Brightness, roughness] deviation (jnd)	[2, 2]	[2, 5]	[5, 2]	[5, 5]
p	0.004	<.001	<.001	<.001
p_adjusted	0.01	0.004	0.002	<.001
cohen-d	0.67	_	0.95	_

S4: effect significance, adjusted significance (Holm corrections for repeating comparisons) and size of t-tests on the response time increases depending on the different singleton brightness values (quantified in jnd) in experiment 5. The shaded columns correspond to conditions for which the distribution is not normal (revealed through a Shapiro test), and for which Wilcoxon tests were applied.
